# Caution ahead: virtual cues for safer wayfinding

**DOI:** 10.1186/s41235-026-00748-4

**Published:** 2026-08-11

**Authors:** Ashley M. Buzard, Yu Zhao, Monika Lohani, Bobby Bodenheimer, Sarah Creem-Regehr, Jeanine Stefanucci

**Affiliations:** 1 Department of Psychology, University of Utah, Salt Lake City, UT USA; 2 Department of Computer Science, Vanderbilt University, Nashville, TN USA

**Keywords:** Virtual cues, Augmented reality, Navigation, Safety

## Abstract

Mobile phones and other smart displays, such as in-car entertainment units, are widely used to display and dictate navigation routes. These navigation systems offer alerts about road closures, construction zones, and traffic conditions. However, these navigation applications often fail to dynamically update safety information in response to rapidly changing threats, such as public safety incidents. The current study explores virtual cues as an intermediate step toward developing augmented reality (AR) safety navigation aids. In a virtual reality (VR) environment simulating a safety–critical scenario, we evaluate two types of navigation cues: (1) a world-fixed localized, egocentric ground-area (GA) cue that visually highlights danger zones and (2) a screen-fixed allocentric minimap (MM) offering a global bird’s-eye view of the danger areas and surroundings. The cues were evaluated using a free-navigation goal directed wayfinding task, in which participants were asked to navigate to a target beacon, answer affective questions providing trial-by-trial evaluation of affective states (i.e., spatial anxiety), and then asked to point to the last known threat location to evaluate spatial memory. Results show both cues were associated with minimal time in danger zones, but the minimap significantly decreased spatial anxiety ratings and increased participants’ reports of willingness to walk the same route in the real world, compared to the GA cue. Further, higher self-reported navigation ability predicted better spatial memory within the virtual environment. These findings represent an initial step toward understanding how virtual cue design affects navigation behavior, affective states, and spatial memory in safety–critical environments.

## Significance statement

Navigation research has largely focused on how virtual graphics can be used to provide directional guidance to navigators, supporting efficient wayfinding in everyday scenarios. Yet real-world navigation can occur in a variety of situations, including public safety emergencies, where individuals must make rapid decisions under increased cognitive load and stress. In these scenarios, directional guidance may not be useful or sufficient to help the navigator reach a point of safety. The current study expands the use case for virtual graphics in navigation by examining their ability to cue hazardous areas in the environment that pose an increased risk to the navigator. This study therefore makes two novel contributions: (1) a systematic evaluation of cues designed specifically to guide hazard avoidance, and (2) a novel methodology for assessing anxiety states around those cues and the situation on a trial-by-trial basis. Together, these extend existing cueing paradigms beyond route guidance, toward the importance of considering safety during navigation, and how individual differences in states and traits shape safe navigation decisions. The results of the study showed that two different virtual graphics promoted safe navigation, but only a minimap cue led to a significant decrease in participants’ state anxiety levels. These results highlight how virtual graphics can serve as intuitive cues to signal dangerous areas in the environment—allowing for safer navigation and reducing momentary state anxiety in safety–critical environments, where rapid, informed decisions are essential.

## Introduction

Spatial knowledge and the ability to adapt navigation strategies in response to navigational challenges are core components of effective wayfinding. Navigation is defined as the process of planning and maintaining a route through an environment. It relies on three different forms of spatial knowledge, including landmark knowledge, route knowledge, and survey knowledge (i.e., knowledge that supports the development of a cognitive map, which is a holistic mental representation of the environment) (Siegel & White, 1975; Werner et al., 1997). Conventional navigation aids (such as smartphones) are effective and convenient for delivering route guidance. However, these systems often fall short in supporting the development of survey knowledge and reorientation skills. This limitation is problematic when navigators face unexpected obstacles or disruptions to their route; in these situations, survey knowledge is necessary for effective navigation. AR is one alternative to conventional navigation aids. By overlaying virtual cues, AR promotes active engagement with the environment that facilitates the development of survey knowledge, reduces time on task, and increases wayfinding efficiency (Bolton et al., 2015; Nurminen & Sirvio, 2021; Zhao et al., 2023). By reducing passive route-following, AR addresses key issues in current GPS text- or map-based navigation aid technologies (Gardony et al., 2021). However, hardware limitations currently hinder the real-world implementation of AR navigation systems. The current study addresses this limitation of AR by evaluating virtual cues in a VR environment as a proof-of-concept. This approach allows us to experimentally evaluate virtual cueing strategies and to take the first step in understanding how virtual cues affect wayfinding patterns, affective states, and spatial memory.

### Virtual cues in navigation

Virtual cues enhance spatial awareness by directly engaging users’ perceptual and cognitive processes, supporting spatial learning and decision-making. Prior research has shown that compared to conventional device-based navigation guidance, visual cues improve orientation, reduce task errors (Kamilakis et al., 2016), and develop survey knowledge (Lakehal et al., 2023; Wenig et al., 2017). A key detail that needs to be considered when developing virtual cues is the frame of reference. This paper focuses on screen-fixed and world-fixed frames of reference because these are most commonly used in navigational cue design. The screen-fixed cues remain aligned with the user’s viewpoint, potentially lowering cognitive load because the navigator does not need to scan the environment in order to see the cue (Baumeister et al., 2017; Zhao et al., 2023). However, screen-fixed cues may also occlude the real world, which can reduce spatial awareness and impede the development of cognitive maps (Kumaran et al., 2023). In contrast, world-fixed cues are anchored in the environment and appear only when the navigator is within a specific proximity or viewing angle of the virtual cue (Zhao et al., 2023). World-fixed cues are presented egocentrically, meaning they are displayed in relation to the navigator’s own position in the environment and appear at spatially relevant positions, so they promote landmark-based navigation and route knowledge (Gardony et al., 2021; Lakehal et al., 2023).

#### Screen-fixed minimaps

Minimaps are one of the most widely implemented screen-fixed cues in virtual environments. They offer a compact, top-down, allocentric perspective of the surrounding environment and are usually embedded within the navigator’s FOV. These cues convey critical spatial information such as the navigator’s real-time location, nearby landmarks, directional goals, and potential obstacles. By supporting survey-level spatial understanding and reducing the need for constant perceptual scanning, minimaps are beneficial in time-sensitive or cognitively demanding tasks (Hsu et al., 2025; Zhang & Nakajima, 2022). When well-designed, minimaps allow users to orient themselves, anticipate navigational decisions, and develop robust cognitive maps (Cao et al., 2024; Mahalil et al., 2019; Qiu et al., 2024).

However, the effectiveness of minimaps depends on visual design displays as well as individual differences. Displays that are overly detailed can introduce visual clutter, which can impede a person’s ability to extract relevant information. In contrast, simplified and clearly designed minimaps have been shown to facilitate faster orientation, better route recall, and lower cognitive load (Zhang & Nakajima, 2022). Similarly, individual differences in spatial ability and spatial anxiety affect the usefulness of minimaps. People with lower spatial ability or higher spatial anxiety may experience difficulty integrating allocentric representations within their egocentric experience, leading to disorientation and cognitive overload in unfamiliar environments (Hsu et al., 2025; Mahalil et al., 2019). Informed by this prior work, we developed a minimap (MM) cue that we expected would both facilitate understanding of dangerous locations and reveal individual differences in effective use and preference.

#### World-fixed cues

In contrast to screen-fixed cues, world-fixed cues are fixed in the environment and offer egocentric visuals aligned to the navigator’s current perspective. This integration can reduce cognitive load, particularly in high-stress situations (Arntz et al., 2020; Bolton et al., 2015; Rubio-Sandoval et al., 2021; Zhao et al., 2023). Directional guidance through arrows, paths, or signage represents typical world-fixed cues, but they can also be designed to communicate other types of spatial information. Prior research shows that egocentric visual cues anchored in the environment can improve situational awareness by drawing attention to key areas, such as landmarks or hazards, without requiring users to interpret abstract symbols (Montuwy et al., 2019; Pivavaruk & Cacho, 2022). Other design elements, such as graphical borders, glow effects, and ground-level highlights, also help delineate navigable versus hazardous areas (Gardony et al., 2021; Kuo et al., 2020; Möller et al., 2012; Thi Minh Tran & Parker, 2020).

In the current study, we build on this work by introducing a world-fixed ground-area (GA) cue designed to highlight dangerous zones rather than provide explicit directional guidance. The GA cue used red and yellow ground highlights to help users recognize and avoid dangerous areas while navigating toward a target beacon. This design approach shifts from route-based to risk-based navigation guidance.

### Virtual cues for safety

Virtual cues have been increasingly explored as a tool to enhance hazard perception, emergency response, and evacuation strategies in high-risk environments using visually salient and intuitive cues (Feng et al., 2020; Kanangkaew et al., 2023; Park et al., 2023; Tang et al., 2009; Zhu et al., 2020). Research in firefighting, search and rescue, and building evacuation has used both screen-fixed and world-fixed virtual cues to convey safety–critical information (Hu et al., 2022; Luksas et al., 2022; Park et al., 2023; Sharma et al., 2020). These cues are designed to direct the users’ attention to key elements in the environment during high-stress and cognitively demanding situations using intuitive and familiar symbols, icons, and bright colors.

In search and rescue missions, virtual cues have been used to enhance ground penetrating radar visualizations, providing first responders with real-time assessments of voids beneath disaster rubble using brightly colored outlines, and helping them in identifying safe extraction paths and assessing structural integrity (Hu et al., 2022). These enhanced visualizations improve rescue coordination by overlaying safe passageways and hazard markers directly onto the environment, allowing teams to operate more efficiently while minimizing the risk of secondary collapses (Hu et al., 2022). Beyond structural assessment, world-fixed localized cues assist in cognitive load management, reducing mental strain in highly dynamic rescue scenarios (Luksas et al., 2022; Smith et al., 2021). In firefighting, world-fixed color-changing virtual trails, which are similar to highlighted paths in navigation research, have been used to reduce search redundancy by changing color from bright green to red to show how long ago an area in a building was searched for the victim (Bailie et al., 2016). World-fixed symbolic icons have also been utilized to represent locations of relevant information in the environment, such as the location of the fire, fire extinguishers, or distances to the nearest exit. In low-visibility, virtual cues become especially useful, and saliently colored arrows or highlights can be used to guide firefighters to trapped individuals (Bailie et al., 2016; Zeman et al., 2022).

Beyond emergency response, virtual cues have also been used in emergency preparedness training. Serious games are commonly used in earthquake simulations to train individuals on evacuation protocols. These systems often use screen-fixed markers like green exit lines and red hazard circles to direct people on the steps of the evacuation and to simulate realistic situations (Feng et al., 2020; Lovreglio et al., 2018). One issue with current evacuation protocols is that buildings use static signage in dynamic emergencies. Mobile AR systems designed for evacuations have used smartphone-guided graphics and dynamic world-fixed signage. Park et al. (2023) developed a mobile AR system with green parallel lines and a central arrow to direct evacuees. Other AR safety systems have used blinking arrows, animated exit signs, and moving door markers, allowing users to evacuate quicker and with fewer errors than when static signs are present (Feng et al., 2020; Lovreglio et al., 2018; Sharma et al., 2020).

While this body of work highlights the promise of virtual navigation aids, most studies focus narrowly on evacuation speed or route efficiency, overlooking factors like cognitive load, affective responses, and spatial memory. To address these gaps in the research, the current study builds on our prior work (Buzard et al., 2023) by introducing a more realistically framed public safety emergency and investigating how virtual cue design impacts navigation behavior, affective state, and spatial knowledge acquisition. Our prior work examined two virtual cues (1) a world-fixed ground-area (GA) cue, and (2) a screen-fixed text cue that showed distance to a threat in meters. While the GA cue effectively guided users to safer routes, the absence of individual affective and cognitive measures made it difficult to disentangle why that cue was superior. The screen-fixed text cue, in contrast, was not effective in alerting users to danger, and participants reported that it obscured information in the FOV. Based on these results, the current study refined the design of the GA cue, and introduced a novel minimap cue that was fixed to the user’s screen and provided real-time safety information.

### Individual differences in navigation

Navigation abilities vary across individuals and can affect route selection, cue interpretation, and spatial strategies (Ishikawa & Montello, 2006; Weisberg & Newcombe, 2016). These individual differences can be understood by evaluating a person’s navigation-relevant traits (e.g., enduring characteristics) and states (e.g., immediate reactions to a specific situation). Spatial ability, spatial anxiety (SA), and harm avoidance (HA) are all trait constructs that could affect a person’s navigation strategy and style. Individuals with higher spatial ability, as measured by the Santa Barbara Sense of Direction (SBSOD) scale (Hegarty et al., 2002), have been shown to favor allocentric, survey-based navigation strategies, while those with lower SBSOD scores favor egocentric, landmark-based navigation strategies. Similarly, spatially anxious individuals utilize more survey-based navigation strategies, and individuals with higher spatial anxiety rely more on landmark or egocentric navigation strategies (Alvarez-Vargas et al., 2020; Hund & Minarik, 2006; Lawton, 1994, 2001). Harm avoidance is a personality trait that reflects a person’s tendency toward caution and risk, and those high in HA often pause, retrace steps, and rely more on explicit cues when navigating (Gagnon et al., 2016).

Although spatial anxiety is often treated as a stable trait, it can fluctuate with task demands. For example, spatial anxiety may spike in response to perceived threats in real-time, even among those low in trait-level measures. Yet, no previous work in the navigation literature, to our knowledge, has examined trial-by-trial changes in *state* spatial anxiety during active navigation. While most of the past work relies on traits, state-level fluctuations could have a significant influence on navigation in complex environments. In the current study, by collecting state-level self-reports of anxiety after each route, our study captures within-subject dynamics that reveal how state-level fluctuations shape cue engagement and performance. In addition to cognitive and emotional traits, prior experience, particularly with video games, shapes spatial ability and navigation behavior. Frequent gaming has been shown to improve spatial memory, reorientation skills, and navigational flexibility in complex environments (Murias et al., 2016; Richardson et al., 2010). These trained skills could buffer against the effects of stress and spatial anxiety by enhancing cue interpretation and adaptability under pressure (Leonhardt & Overå, 2021).

The individual differences discussed above underscore the need for navigation cues that help to reduce anxiety in wayfinding. To account for individual differences in our study, we included validated trait measures: the Santa Barbara Sense of Direction scale (Hegarty et al., 2002), the Lawton Spatial Anxiety Scale (Lawton, 1994), the Tellegen Harm Avoidance subscale (Tellegen & Waller, 2008), and self-reported weekly gaming hours. We also collect route-level state responses via verbal Likert ratings, offering insight into how emotional and cognitive states evolve during navigation and shape cue engagement in real-time.

### Current study

The current study investigates how AR-relevant virtual, visual cues can enhance spatial memory, reduce anxiety, and guide safer wayfinding in safety–critical contexts. We employed two distinct cues and examined their influence on guiding safe wayfinding, enhancing spatial memory for threat locations, and reducing a participant’s anxiety. The cue effects were examined via a wayfinding task where users were asked to navigate to a target beacon in the safest and most efficient manner. Once participants reached the target beacon, they answered questions about their state spatial anxiety and subjective performance. After answering the questions, participants pointed to the remembered threat location, and provided a confidence judgment about how accurately they pointed to the threat before progressing to the next trial. Safe navigation in the task was defined to the participant as limiting their time in danger zones, which meant that they were out of range from an active shooter at the threat location and would be safe. Specifically, we examined two cueing strategies: (1) a world-fixed ground-area (GA) cue that visually highlighted danger zones within the user’s field of view (FOV), reinforcing local landmark engagement since the participant was able to use surrounding buildings to understand where the threat was in the environment, and (2) a screen-fixed minimap (MM) cue that provided a continuous, allocentric overview (which is a map-based bird’s-eye perspective) of danger zones and alternate paths, offering a global perspective. We predicted that the GA cue would be more effective in guiding safe navigation, but that the MM cue would reduce state anxiety and improve spatial memory for the threat location.

We measured three primary dependent variables—time spent in danger zones, state spatial anxiety, and spatial knowledge (pointing accuracy). We also examined whether spatial anxiety and confidence measured at the state level (on each trial) related to trait-level measures. Finally, we measured cue preference and whether this varied by gender. The following sections give our hypotheses for each of these variables.

#### Time in danger zones

Participants were asked to navigate to a target as safely and efficiently as possible, which implied avoidance of threats. We predicted that participants using the world-fixed GA cue would spend less time in danger zones (TID) than those using the screen-fixed MM cue **(H1)**. The GA cue displays information egocentrically and presented the danger zones directly in the participant’s FOV, which should eliminate the need for spatial transformations to understand the location of the threat, allowing for more precise judgments of where the danger zone is located. Because video games often use minimaps and train spatial transformation skills (Murias et al., 2016; Richardson et al., 2010), we predicted that participants with more weekly gaming hours would spend less TID when the MM cue was present, but that video game hours would not influence TID for the GA cue **(H1a)**. Finally, we predicted that higher levels of trait SA and HA would be associated with decreased TID **(H1b)** because trait SA and HA are both linked to more cautious navigation behavior (Gagnon et al., 2016; Lawton, 2001).

#### State spatial anxiety

The MM cue offered a global, persistent, allocentric bird’s-eye view of the environment; we predicted that participants would feel less spatially anxious while using it compared to the GA cue **(H2)**. Although the GA cue provides local, direct, egocentric feedback about safety, it requires more active scanning and interpretation within the FOV to understand threat location, which may increase uncertainty or SA. We also predicted that state-level SA would increase with exposure to the danger zones and that longer exposure would correspond to higher SA ratings at the end of a route **(H2a)**, suggesting that even virtual threat exposure can heighten affective responses.

#### Spatial knowledge

Given that the MM cue provides a continuous allocentric view of the environment, it supports the development of survey-based knowledge. As a result, we predicted that given participants had continuous access to survey information and would be able to develop survey knowledge more easily as they navigated, participants using the MM cue would be more accurate at pointing to prior threat locations than those using the GA cue **(H3)**. We also predicted that participants with higher SBSOD scores would have better spatial memory in the VE, which would be reflected in lower pointing errors, compared to those with lower SBSOD scores **(H3a)**. In addition, we predicted that participants’ confidence in their pointing accuracy would increase over time **(H3b)**, reflecting task learning and growing familiarity with both the environment and the demands of the pointing task.

#### Trait and state relationship

We predicted that participants’ reported state-level spatial anxiety and pointing confidence would mimic their trait-level tendencies. In other words, participants with high trait SA would also report feeling more spatially anxious during the VR task **(H4)**, and those with higher SBSOD scores would report greater confidence in their pointing responses **(H4a)**. Such a trait–state alignment would support the idea that VR allows for a meaningful evaluation of individual differences in spatial anxiety.

#### Gender differences in cue preference

We predicted a gender difference in cue preference, with males preferring the MM cue **(H5)**. This prediction is based on prior work showing that males tend to favor survey-based, allocentric navigation strategies, which aligns with the MM’s cue presentation style, while females typically prefer more landmark and route-based navigation strategies (Lawton, 1996; Sandstrom et al., 1998).

## Method

### Participants

Sixty-eight participants (42 females, 26 males) were recruited from the undergraduate psychology participant pool and through community recruitment. Participants were compensated with either course credit or $10 for their participation. Six participants’ (4 females, 2 males) data were excluded due to technical issues, and three participants (3 females) withdrew due to cybersickness. This resulted in a final sample of 59 participants (35 females, 24 males; *M* = 20.59 years, SD = 3.74).

The sample size was determined through a power analysis, using an effect size of 0.40 (based on our prior related work) (Buzard et al., 2023), an alpha level of 0.05, and a desired power of 0.80. Results indicated that a minimum of 52 participants would be required to detect the main effects of cue type on safe navigation. All participants had normal or corrected-to-normal vision. This study was approved by the University of Utah Institutional Review Board (IRB). Study questionnaires were administered and managed using REDCap electronic data capture tools hosted at the University of Utah. REDCap (Research Electronic Data Capture) is a secure, web-based software platform designed to support data capture for research studies (Harris et al., 2009, 2019).

### Apparatus

The study was conducted using the Varjo VR-3 head-mounted display (HMD), which features human-eye resolution, a 90 Hz frame rate, an ultra-low latency at less than 20 ms (which is below the threshold of delay that humans can perceive), and a 115° × 90° FOV. The HMD weighs approximately 980 g and automatically adjusts the interpupillary distance (IPD) for users within its 59–71 mm range during the initial calibration sequence. Participants in this study had a mean IPD of 62.49 mm (SD = 3.42 mm), with 10 individuals falling below 59 mm and none exceeding 71 mm. Manual adjustments were made to the closest supported value for participants whose IPD fell outside the supported range (e.g., 59 mm).

In the study, participants were seated in a swivel chair and used body-based locomotion to control their movement in the VE. A Vive Tracker (version 3.0) was secured to an adjustable belt around the waist to track body rotation, which allowed participants to control their heading in the VE by physically turning their bodies in the real world. Forward movement was initiated by touching the top of the trackpad on a VIVE controller, which was held in the dominant hand (see also (Zhao et al., 2023)). The movement speed was instantaneous and fixed at 3 m/s. Participants could only move forward in order to reduce the possibility of developing cybersickness. If the participant wished to turn around in the VE, they needed to turn their body 180^*◦*^ in the real world.

### Virtual environment

The VE was developed using Unity (version 2020.3.20f1) and depicted a large urban city environment designed with an eight-by-ten grid layout, which included approximately 8800 square meters of navigable space. The city consisted of main roadways that were interconnected by alleyways, which provided multiple paths to the target beacons. A predetermined continuous route through the city was divided into twelve route segments, each ending at a beacon that served as both the endpoint of the current segment and the starting point for the next. The target beacon was displayed as a world-fixed cue, and appeared visible when it was within the line of sight of the participant. The beacon floated above the ground and was situated above the halo marker that marked the end of that route segment. Each route segment had one designated threat building, which was outlined in red (regardless of the safety cue presented for that route) to indicate where the active shooter was located. The danger zones were determined using a 100° viewing angle from the top of the building that was aligned with the building’s geometry. The red zone (high danger) extended up to 30 m from the building, and the yellow zone (moderate danger) extended up to 60 m. The routes had an average length of 256 Unity meters (SD = 10.2), and participants took an average of 147.25 s to complete the routes (SD = 51.7).

### Virtual cues

Within the FOV of the worn HMD, participants could see the threat building (outlined in red), the safety cue, a trial counter that displayed the number of completed routes and how many were left to complete (in the top center), and a timer (in the top center) that was used to incentivize safe and efficient navigation (see “Procedure” for additional detail).

**Fig. 1:**
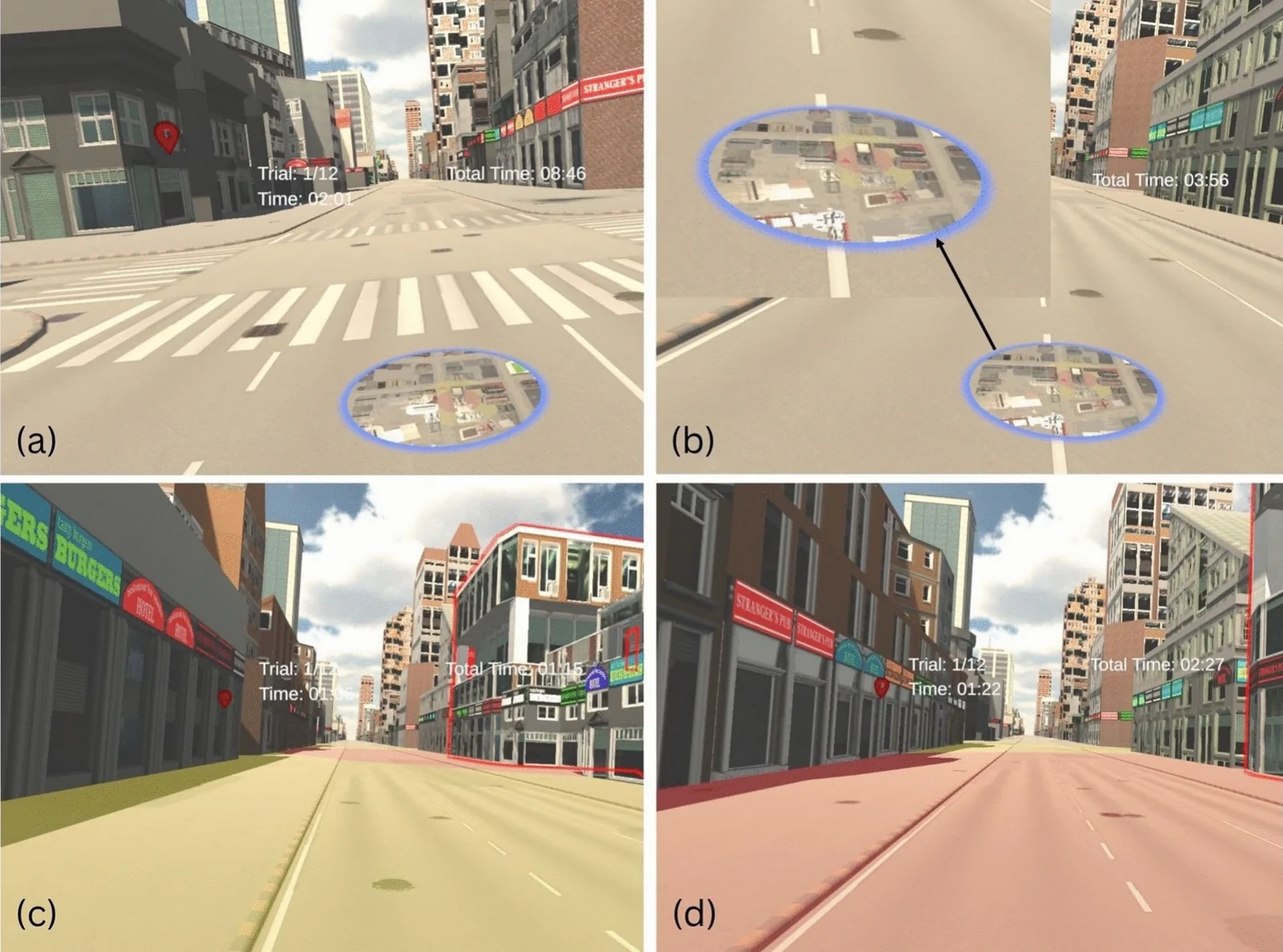
The figure above shows the two cues in the current study along with how they signaled varying levels of danger: **a** minimap (MM) cue showing the participant in a moderate danger (yellow) zone, **b** a zoomed in perspective of the MM cue showing a participant in the high-danger (red) zone, **c** ground-area (GA) cue showing the participant in a moderate danger zone, **d** GA cue showing the participant in a high-danger zone

Two virtual cues were employed to indicate where the danger zones were within the VE (see Fig. 1). The GA cue was a world-fixed cue that highlighted the dangerous zones directly on the ground, with red highlights marking high-danger areas and yellow highlights indicating moderate danger. The GA cue was only visible when the threat building was within the participant’s line of sight, and it was designed to be transparent so that the participant could still see the VE underneath it. In contrast, the MM cue was a screen-fixed cue that displayed a 2D overhead view of the virtual world covering a 150-m radius around the user’s position at all times. The MM cue displayed the danger zones in the same manner as the GA cue, with the danger zone becoming visible when it was within 150 m of the participant. The MM cue was always visible within the participant’s FOV (in the lower left corner). The MM cue displayed an updating red arrow icon to show participants their real-time location within the VE, which ensured continuous access to relative safety information regardless of line of sight.

### Procedure

After giving consent, participants completed an initial questionnaire, which collected self-reported demographics, weekly video game hours, and responses to the SBSOD. Upon completion, participants then proceeded to the VR task, where they completed two practice trials to familiarize themselves with each type of safety cue and the locomotion method before the wayfinding task began. During the practice trials, participants received standardized instructions explaining that the virtual cues signaled danger zones within the environment (indicative of an active shooter located in the cued building) and that they should use the cues to navigate safely while also reaching the target beacon as quickly as possible.

Participants were told that extended time in danger zones was considered unsafe because they would be located within the range of the shooter. They were also made aware that the timer in their FOV would increase its speed within yellow and red danger zones (three seconds would be added for every second spent in the yellow zone, and five seconds added for every second spent in the red zone), simulating a time penalty akin to a health bar in video games. Participants were told that if they finished the task within 25 total minutes (including penalty time), they would receive bonus compensation. In actuality, all participants received the bonus.

**Fig. 2:**
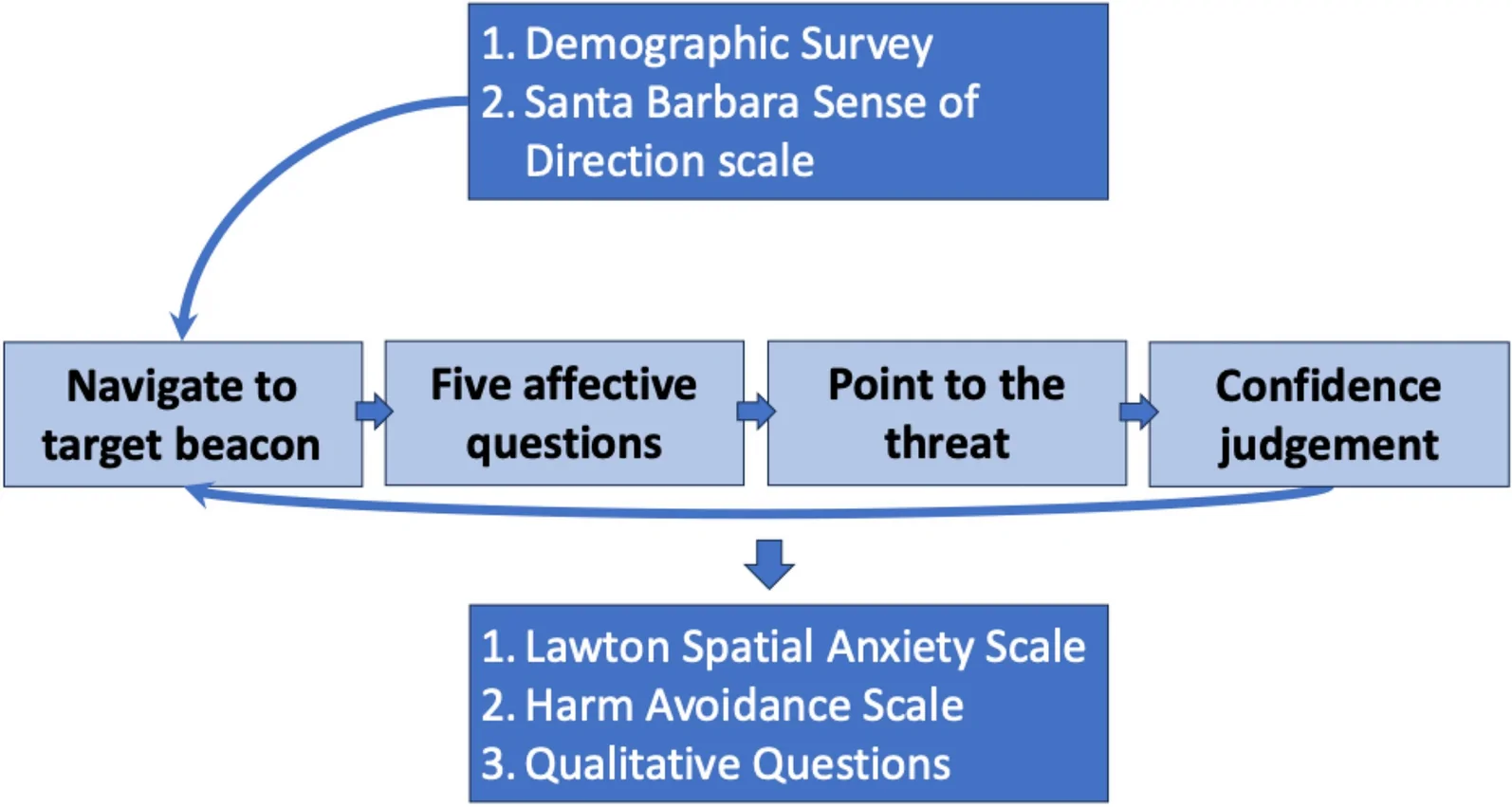
The experimental procedure is outlined above. The boxes in light blue are repeated for all 12 routes, with cue type alternating trial-to-trial

Following the practice trials, participants completed the 12 experimental routes. To control for potential order effects, participants were pseudo-randomly assigned to one of two groups. Odd-numbered participants began the routes with the GA cue, while even-numbered participants started with the MM cue. All participants experienced both cues with the cue type alternating trial-to-trial. When a route was completed, the participant was instructed to hold their head still, and all virtual cues were removed from the environment. Participants then verbally responded to a series of five questions: (1) "How anxious did you feel while navigating the route?", (2) "How threatened did you feel while navigating the route?", (3) "How helpful was the cue in avoiding the danger?", (4) "If this were a real-world situation, how likely would you be to take that same route?", and (5) "Did you ever feel lost or disoriented during the route?". The first four questions were recorded on a 0–10 Likert scale, and the response to question 5 was recorded as yes/no. After responding to the five questions, participants performed a spatial memory task in which they pointed to the last known threat location using a VIVE controller. During this portion of the study, a straight ray cast appeared from the top of the VIVE controller, enabling participants to see where they were pointing before making their selection. When the trigger button was pressed (indicating the spot where the participants believed the threat had been located), the system recorded the estimated pointing direction and the actual ground-truth direction along the *x*–*z* plane, which was used to calculate pointing error (ranging from 0° to 180°, with smaller values indicating higher accuracy and stronger spatial knowledge). Participants then verbally rated their confidence on a 0–10 scale by answering the question, "How confident do you feel that you accurately pointed to where the threat was located?". This response was used in later analyses to examine the relationship between real-world and virtual perceived spatial ability (Fig. 2). After completing all routes, participants did post-study questionnaires, which included the Lawton Spatial Anxiety (SA) scale (Lawton, 1994), the Harm Avoidance (HA) scale (Tellegen & Waller, 2008), and additional qualitative questions assessing cue preference, perceived navigational confidence, disorientation, and anxiety related to the danger zones. These responses provided further insights into how participants perceived their virtual navigation abilities in relation to real-world navigation and whether the virtual cues influenced their route selection and emotional state.

## Results

### Time in danger zones

To examine the effects of virtual cue type and individual differences on participants’ time spent in danger zones, we ran a linear mixed-effects model using R (version 4.3.3) and the *lme4* package (Bates et al., 2015). The model included fixed effects for cue type (GA vs. MM), harm avoidance (HA), trait spatial anxiety (SA), weekly gaming hours, and gender. Random effects were included for participants and routes to account for individual variability. The mean TID for the GA cue was 7.21 s (SE = 1*.*99), and the mean TID for the MM cue was 8.37 s (SE = 0*.*92).

We tested **H1** and found that cue type (GA vs. MM) was not a significant predictor of time spent in danger zones (TID), (*β* = 1*.*16, SE = 0*.*92, *t*(702) = 1*.*26, *p* = *.*21, *d* = 0*.*10). We tested **(H1a)**, and found that contrary to our hypothesis weekly gaming hours did not significantly predict TID (*β* = *− *0*.*48, *SE* = 0*.*41, *t*(704) = *− *1*.*16, *p* = *.*23, *d* = 0*.*04). The interaction between cue type and weekly gaming hours was also non-significant (*β* = *− *0*.*108, SE = 0*.*198, *t*(704) = *− *0*.*55, *d* = 0*.*01, *p* = *.*58). However, it is important to note that most participants reported only 0–2 gaming hours per week (*M* = 2.45, SD = 4.70). Individual differences in trait SA (*β* = *− *2*.*38, SE = 1*.*72, *t*(704) = *− *1*.*38, *p* = *.*17, *d* = 0*.*19) and trait HA (*β* = 0*.*55, SE = 0*.*39, *t*(704) = 1*.*40, *p* = *.*16, *d* = 0*.*05) also did not predict time spent in danger zones, which was counter to **(H1b)**.

To further examine whether the non-significant effects reflected true null results or were due to insufficient power, we conducted Bayesian model comparisons using the BayesFactor package (Morey et al., 2023). The full model included fixed effects for cue type, HA, SA, and weekly gaming hours, with random effects included for participants and routes. Compared to a reduced model excluding cue type, the *Bayes Factor* indicated moderate-to-strong evidence against including cue as a predictor (*BF* = 0*.*18). This result confirms that there was no meaningful difference between the two cue types tested in TID. Bayesian model comparison is particularly valuable in this context because, unlike null hypothesis significance testing, Bayes factors can quantify evidence in favor of the null model (Wagenmakers et al., 2018). In contrast, removing HA (*BF* = 0*.*93) or SA (*BF* = 0*.*90) resulted in negligible changes in model fit, indicating inconclusive evidence for or against their inclusion. Weekly gaming hours showed anecdotal support for inclusion (*BF* = 1*.*28). The results indicate that while the absence of a cue effect is supported across both analyses, the null findings for individual differences are best interpreted as inconclusive.

Additionally, to rule out potential learning effects from the within-participants design, we conducted a separate repeated-measures multilevel model that included cue order. Cue order did not significantly predict TID (*p* = *.*70), suggesting that counterbalancing was successful and performance was not meaningfully affected by which cue was presented in the first trial. The lack of effects for TID led us to conduct a positional data analysis, using the x and z coordinates of participant movement, to evaluate if there were other behavioral differences between the two cue types that TID was not capturing. We found that the MM cue led to faster route completion times (*M* = 10.03 s) and shorter pausing durations (*M* =.36 s) than the GA cue. There was no difference in the number of pauses taken by a participant between the MM and GA cues. This suggests that participants made the same number of navigational decisions while pausing but spent less time deliberating at decision points, a factor that could be important in safety–critical navigation scenarios.

### State spatial anxiety

In this analysis, we tested how cue type and time in danger zones influenced participants’ state SA as the dependent variable **(H2)**, using a multilevel model with random intercepts for participant and route. This analysis tested our prediction that the MM cue would reduce feelings of state SA compared to the GA cue. State SA was computed as the average of participants’ responses to two questions asked at the end of each route: (1) “How anxious did you feel while navigating the route?” and (2) “How threatened did you feel while navigating the route?”.

**Fig. 3:**
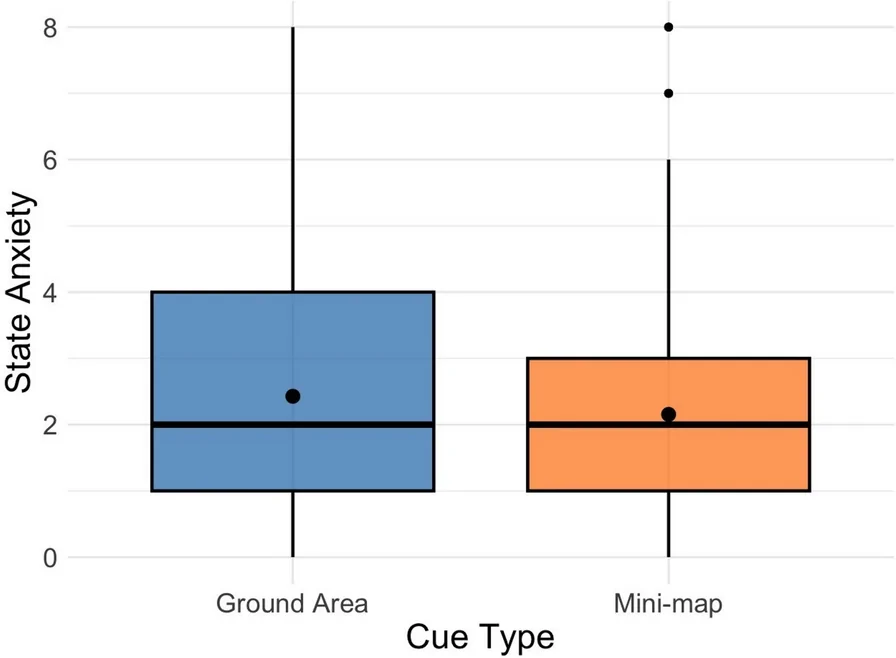
State anxiety ratings were significantly lower when participants navigated with the minimap (MM) cue than with the ground-area (GA) cue, though the effect was small (*d* =.19). In the figure above, the median is represented by the horizontal line, and the mean is represented by the black dot

Consistent with **H2**, we found that cue type significantly predicted state SA, with participants reporting lower state SA when using the MM cue to navigate compared to the GA cue (*β* = *− *0*.*28, SE = 0*.*11, *t*(704) = *− *2*.*55, *p* = *.*011, *d* = 0.19), see Fig. 3. We also explored whether time spent in danger zones predicted higher state SA **(H2a)** and found support for this hypothesis as time spent in danger zones did significantly predict increased state SA ratings (*β* = 0*.*009, SE = 0*.*004, *t*(706) = 2*.*32, *p* = *.*021, *d* = 0*.*01). In addition to cue type and time in danger zone effects on state SA, we also tested whether state SA fluctuated across the 12 routes. Results showed that while there were clear between-participant differences in reported state SA, these ratings did not vary significantly across routes (*β* = 0*.*01, SE = 0*.*01, *t*(706) = 1*.*20, *p* = *.*230, *d* = *.*01), suggesting that the routes did not produce varying levels of state feelings of SA.

Further, exploratory analyses were conducted to better understand how state SA and cue type influenced the answer to question 4 ("If this were a real-world situation, how likely would you be to take that same route?”). A linear mixed-effects model was fit. There was a significant negative relationship between state SA and real-world willingness (*β* = *− *0*.*53, SE = 0*.*054, *t*(706) = *− *9*.*75, *p* < *.*001, *d* = *− *0*.*30), indicating that participants who felt more spatially anxious during a given route were significantly less likely to report willingness to walk that same route in a real-world setting. Cue type also influenced route willingness, with participants more likely to walk the same route in a real-world scenario when the MM cue was present compared to the GA cue (*β* = 0*.*29, SE = 0*.*13, *t*(706) = 2*.*25, *p* = *.*024, *d* = 0*.*17).

### Spatial knowledge

To test **H3**, we analyzed participants’ pointing accuracy as the dependent variable using the angular error that was recorded after each route. A linear mixed-effects model was fit. We predicted that participants navigating with the screen-fixed MM cue would exhibit more accurate spatial memory, i.e., lower pointing error, compared to those using the world-fixed GA cue. Contrary to expectation, **H3** was not supported, and cue type did not significantly predict pointing error (*β* = 1*.*14, SE = 1*.*11, *t*(706) = 1*.*03, *p* > *.*05, *d* = 0*.*08). However, overall pointing accuracy was high across all participants (*M* = 14.46°, SD = 10.12°), which may have limited our ability to detect cue-related differences in spatial memory. To assess whether the null effect of cue type on pointing error reflected a true absence of effect or insufficient power due to ceiling performance and limited variability, we conducted a Bayesian model comparison. Using the *BayesFactor* package (Morey et al., 2023), the model including cue type was compared to a null model with only random effects for participants and routes. The Bayes factor indicated moderate-to-strong evidence against including cue as a predictor of pointing error (*BF* = 0*.*14), supporting the null result observed in the frequentist analysis.

**Fig. 4:**
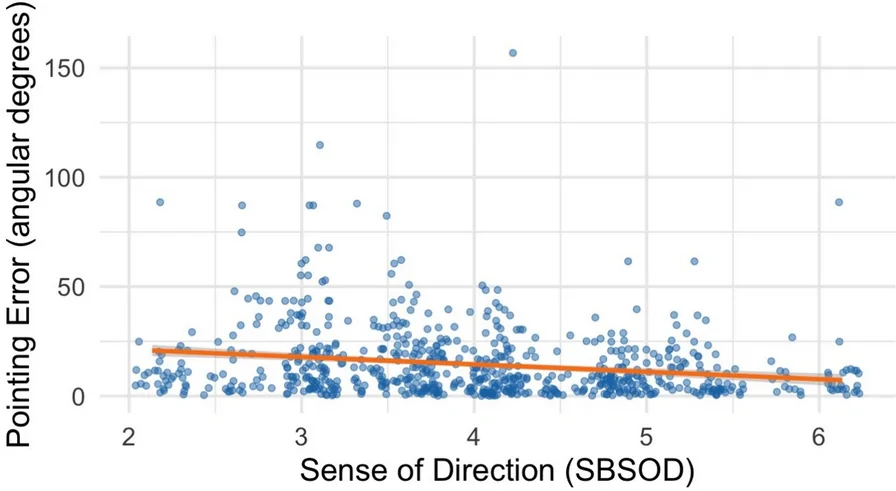
Higher self-reported sense of direction, as measured by the SBSOD scale, was associated with significantly lower pointing error, although the effect was small (*d* = 0.23). The orange line represents the linear trend, and each point represents a single trial from a single participant

**Fig. 5:**
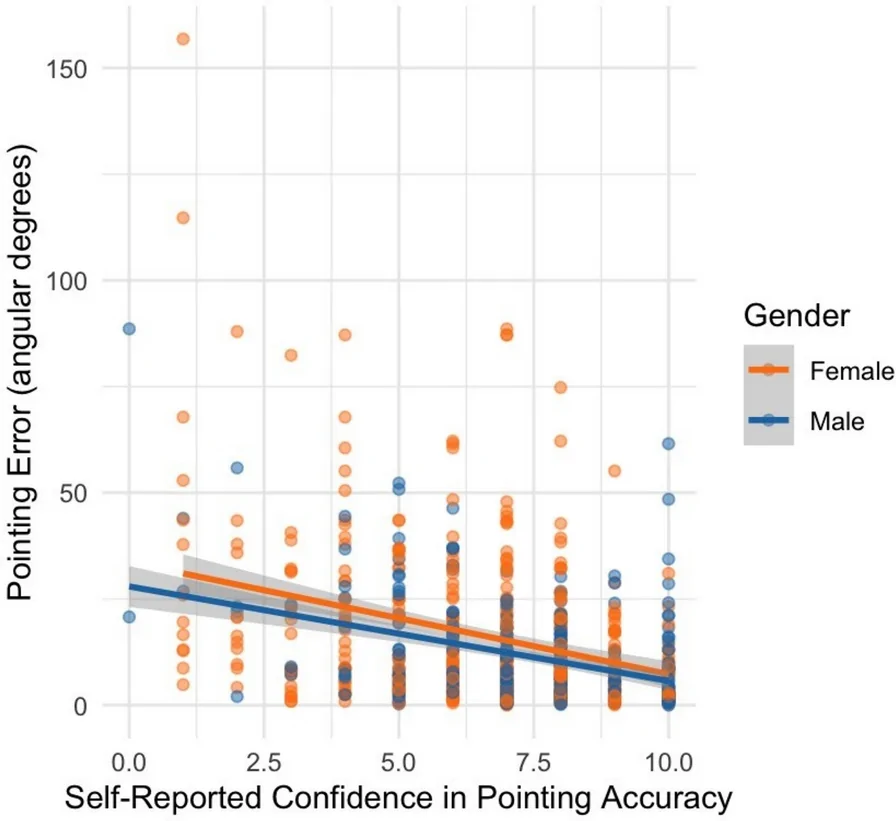
Greater self-reported confidence in pointing accuracy was associated with significantly lower pointing error for both males and females, although the effect was small (*d* = 0.17). Each point represents a single trial from a single participant, and shaded regions represent the 95% confidence interval around each regression line

We did find support for **H3a**, revealing that self-reported spatial ability was related to participants’ pointing errors. Specifically, participants with higher spatial ability (as assessed via the SBSOD) exhibited significantly lower pointing errors (*β* = *− *3*.*39, SE = 0*.*94, *t*(706) = *− *3*.*62, *p* < *.*001, *d* = *− *0*.*23). This suggests that self-reported spatial abilities in the real world translate to spatial abilities in virtual simulations (see Fig. 4). Hypothesis **H3b** was not supported as no significant linear trend in confidence ratings over time was found (*β* = *− *0*.*01, SE = 0*.*02, *t*(706) = *− *0*.*45, *p* = *.*652, *d* = 0*.*01). However, exploratory analysis revealed that pointing confidence did significantly predict pointing accuracy, with greater confidence associated with lower pointing error for both males and females (*β* = *− *2*.*46, *SE* = 0*.*29, *t*(706) = *− *8*.*58, *p* < *.*001, *d* = *− *0*.*17), see Fig. 5. Gender differences also emerged in our exploratory analyses, with males reporting significantly higher confidence in their pointing than females (*β* = 1*.*00, SE = 0*.*45, *t*(706) = 2*.*23, *p* = *.*028, *d* = 0*.*58) and demonstrating significantly lower pointing error (*p* = *.*018), with an average increase in accuracy of 4.74^*◦*^. There was no interaction between route and gender, (*β* = *− *0*.*03, SE = 0*.*04, *t*(706) = *− *0*.*87, *p* = *.*383, *d* = 0*.*02), indicating that confidence levels across routes were similar for both males and females.

### Trait and state relationships

To better understand how stable, trait-level individual differences related to participants’ real-time, state experiences in VR, we conducted Pearson correlation analyses (corrected for multiple comparisons) between trait-based measures (SBSOD, SA) and their corresponding state-level indicators. Consistent with **H4**, we observed a significant correlation between trait-level and state-level SA (*r* (57) = *.*30*, p* = *.*021) suggesting that individuals who tend to experience higher trait SA also reported greater state SA during the virtual navigation task. This finding indicates that trait SA meaningfully relates to the real-time affective experience of navigating in a VE, at least for the current task.

To assess state and trait spatial ability, we compared participants’ trait SBSOD scores (Hegarty et al., 2002), which reflect self-reported, real-world spatial ability, with a VR-specific state measure of perceived spatial ability. After each route, participants rated their confidence in their pointing response to the last known threat location by answering: “How confident are you that you pointed accurately to the last known threat location?". The answer to this question was used to determine if a person self-reports spatial ability in the same manner in both real-world scenarios and in a VE. The results showed a significant positive correlation between trait-level and state-level spatial ability in VR (*r* (57) = *.*44*, p* < *.*001), indicating that participants who report having stronger trait spatial abilities were also more confident in their spatial ability within the VE. These findings support **H4a** and the use of SBSOD as a meaningful individual difference measure in VR navigation studies and suggests that perceived spatial ability is relatively stable across trait and state reports of ability for this task. Overall, these findings support, trait SA, and self-reported spatial ability meaningfully translate to participants’ affective responses and self-evaluations in VR.

### Gender differences in cue preference

To test **H5**, we conducted a Chi-square test of independence between gender and cue preference. The analysis revealed no significant relationship (*χ*^2^(1) = 0*.*55, *p* = *.*46, *ϕ* = *.*10); male and female participants were equally likely to prefer the MM or GA cues. However, when examining overall cue preference across all participants, a significant preference for the MM cue emerged (*χ*^2^(1) = 16*.*29, *p* < *.*001, *ϕ* = *.*53). This finding suggests that, regardless of gender, participants favored the screen-fixed, allocentric MM cue over the world-fixed GA cue for navigating in the current safety–critical environment. **H5** was not supported, but the strong overall preference for the MM cue reinforces the potential value of minimaps as intuitive, user-preferred navigation tools.

### Perceived cue effectiveness and disorientation

To further explore cue effectiveness, we examined participants’ ratings of how helpful each cue was for avoiding the danger zones. Participants answered the question,

"How helpful was the cue for allowing you to avoid the threat?" and provided a rating on a Likert scale from 0 to 10 at the end of each route. Cue type, GA or MM, and route were used for this analysis. Participants rated the MM cue as significantly more helpful than the GA cue (*β* = 1*.*27, *SE* = 0*.*15, *t*(705) = 8*.*42, *p* < *.*001, *d* = 0*.*63). Perceived helpfulness did decline slightly over time (*β* = *− *0*.*07, SE = 0*.*02, *t*(705) = *− *3*.*26, *p* = *.*001, *d* = 0*.*04), which suggests that participants may have developed internal strategies as routes progressed. Alternatively, they reduced their reliance on the virtual cues as their familiarity with the VE increased. Participants were also asked, "Did you ever feel lost or disoriented on the route?" and gave a yes/no verbal response. A logistic regression was conducted to examine the effects of cue type on ratings of feeling lost or disoriented. Participants were significantly less likely to report feeling lost when navigating with the MM cue compared to the GA cue (*β* = *− *0*.*43, *p* = *.*015, OR = 0.65, 95% CI [0.46, 0.92]). However, over the course of the task, feelings of disorientation declined (*β* = *− *0*.*09, *p* < *.*001, OR = 0.91, 95% CI [0.87, 0.96]), which is consistent with a learning effect as participants became increasingly familiar with the environment and task structure.

## Discussion

This study examined how virtual cues to develop safety navigation aids (i.e., a ground-area cue of threat versus a minimap of the environment) can influence navigation safety, spatial knowledge development, and affective states in a large-scale urban VE. The study expanded upon prior work by introducing a trial-by-trial evaluation of affective states and demonstrated the importance of considering individual affective differences when designing and testing navigational cues for safety. Although the two cue types did not differ for time in danger zones, there was a significant difference in the cues’ influence on affective states. The MM cue led to a significant decrease in anxiety, which potentially allowed participants to better orient themselves in the VE and navigate efficiently (Varshney et al., 2024). A major aim of this study was to understand whether cue type influenced participants’ time in danger zones. Our results showed that cue type did not significantly predict TID and that both the MM and GA cues were equally effective in helping participants to avoid the danger zones, since overall participants had very low TID, thus supporting safer navigation. One possible explanation for this lack of an effect—and a general tendency toward completely avoiding dangerous areas—is that both cues were designed to be visually intuitive and provide clear information about where the danger zones were located. Because of the clear and easy-to-interpret design, participants may have been able to use both cues equally well to avoid walking into dangerous areas. We did not include a control condition where no threat cues were provided, so we cannot make strong claims about the amount of improvement over the absence of cues, only that both of the cues used here supported safe navigation behavior. Our prior work has demonstrated an advantage for a ground-area cue over a text-based cue providing information about distance to the dangerous areas, suggesting that different cues (or no cues) can have differential benefits (Buzard et al., 2023). Additionally, positional data analysis showed that although the cues facilitated the same safe navigation behavior and led to successful avoidance of the danger zones, the MM cue did reduce the duration that participants paused and led to a slight decrease in average route completion times. This suggests more confident decision-making with the MM cue. Given the similar safety performance between cues, future work should work to better understand this relationship and whether the MM cue continues to reduce pausing behavior in more complex environments, as this may have benefits for navigation in safety–critical scenarios, where quick, confident decisions may be necessary.

Participants did report significantly less state spatial anxiety when using the MM cue, although the effect was small (*d* = 0.19), see Fig. 3. This finding suggests that while both cues were effective, the MM cue aided in a better sense of control than the GA cue, which required continuous scanning of the environment to understand its extent and boundaries. In safety–critical scenarios, even a small reduction in anxiety could support more confident decision-making, though future work is needed to determine whether this translates to practically meaningful differences outside of a controlled VR environment. However, we do not want to overstate the benefits of the MM cue. It did reduce spatial anxiety in the current task, but the continuous access to survey information provided by this cue may introduce potential trade-offs in real-world situations that have high task demands. For instance, in complex environments, minimaps could increase cognitive load (Hsu et al., 2025; Kumaran et al., 2023; Zhang & Nakajima, 2022). Additional research should examine the utility and cognitive costs of MM cues across many safety–critical contexts.

### Individual differences affecting performance

This study examined whether individual differences in weekly gaming hours, spatial anxiety, harm avoidance, and spatial ability influenced navigation behavior in a safety–critical scenario. To our knowledge, this is the first time that these factors have been taken into consideration when analyzing cues for safety–critical scenarios. Although prior research has found that gaming experience does impact navigation performance in VR (Leonhardt & Overå, 2021; Murias et al., 2016; Richardson et al., 2010), the current study did not find a significant relationship between self-reported weekly gaming hours and TID. However, this non-significant finding may reflect a floor effect in TID or limited variance in gaming hours within our sample to detect a potential effect, given that most participants reported a low number of gaming hours per week. Trait spatial ability and harm avoidance were also not significant predictors of TID. The lack of significant trait effects suggests that real-time strategies and other cognitive factors (i.e., prior experience) may outweigh trait tendencies during safety–critical navigation, and further research is needed to better understand this relationship. Additionally, state spatial anxiety did not increase with TID but was related to which cue was presented for the route, suggesting that reports of state spatial anxiety were more influenced by perceptions of the cue’s effectiveness rather than actual exposure to a threat. In this scenario, the MM cue led to a greater decrease in reports of state SA, showing that in safety–critical scenarios, participants may feel more confident about their navigation decisions and less anxious when presented with wayfinding guidance through a screen-fixed allocentric viewpoint instead of a world-fixed egocentric viewpoint. This finding suggests that participants value having more information available to them in these types of navigation situations. Further, even though the MM cue introduces more visual clutter, it was beneficial for reducing anxiety. Taken together, the results suggest that it is important to consider individual differences when designing cues for navigation, as these factors can significantly impact how individuals interact with cues. It also shows that when designing navigation cues, researchers need to consider the use scenario, as different cues are beneficial for different tasks, and there is no one-size-fits-all model.

### Spatial knowledge development

To evaluate spatial memory, which has not been done in prior research in safety–critical navigation scenarios, we measured participants’ angular pointing errors when recalling the last known threat location to assess how cue type influenced spatial knowledge. Contrary to our hypothesis, cue type did not significantly predict pointing accuracy. We expected that participants using the MM cue would demonstrate better spatial memory due to the access of survey knowledge while navigating, which has been shown to promote cognitive map development (Ishikawa & Montello, 2006; Weisberg, 2014; Werner et al., 1997). However, in the current study, participants showed similar levels of spatial memory development across both cue types, and overall, had high pointing accuracy. One potential explanation for this finding is that during threatening scenarios, when it is beneficial for safety, individuals have better spatial memory. Prior research has shown that acute stress experienced during encoding can improve memory for specific items or events (Mather & Sutherland, 2011; Shields et al., 2022; Zsidó, 2024). Therefore, it is possible that participants felt more acute stress because of the saliency of the threat, which led to better memory of the threat location and perhaps better spatial memory overall of the environment. The overall high accuracy and low angular pointing error showed that participants were encoding where the threat was and were able to recall it when asked at the end of the route, regardless of cue type. The pointing task, therefore, also acted as a manipulation check and allowed us to conclude that participants were actively trying to avoid the danger zones, and that the overall low TID was not just due to wandering or low levels of involvement.

Another explanation for this result could be that although angular pointing error is commonly used to assess spatial memory, it may not fully capture spatial knowledge in small-scale environments where distance-based discrepancies could be more informative. Because we did not control for the distance between participants’ final position and the actual threat location on each route, similar angular errors may still reflect differing spatial accuracy. The factors discussed above could have led to the low overall pointing error we observed in comparison with much of the prior work (Weisberg et al., 2018; Yüksel et al., 2025; Zhao et al., 2023) on pointing measures for spatial memory (where pointing error is usually around 20–30 degrees, at least). Because the error was so low across all participants, any potential effects of the cues may not have emerged due to the limited variance to explain.

The current study did find that individual differences played a role in pointing accuracy. Participants who reported being more confident in where they pointed were significantly more accurate. Additionally, participants who self-reported higher spatial ability on the SBSOD questionnaire were significantly more accurate in pointing back to the last threat location. These effects were observed across both genders. This strong correlation among individuals’ perceived confidence, self-reported spatial abilities, and actual performance is an important finding that further supports VR’s value as a tool for spatial skill evaluation and training in research and applied settings. In applied settings, the current study shows that VR simulations can be used to support spatial memory. Further work could adjust system feedback according to an individual’s own needs and self-perceived ability or the demands of the current safety task.

#### State versus trait self-report measures

The results of the current study validated spatial ability and spatial anxiety as meaningful constructs in VR research and training applications, with a strong alignment between participants’ traits and their self-reported states in VR. Trait spatial anxiety positively correlated with state spatial anxiety, which reinforced VR’s ability to evoke ecologically valid affective responses in safety–critical simulations. This finding is also consistent with prior work showing that trait spatial anxiety predicts performance across both real and virtual contexts (Stachoň et al., 2025; Varshney et al., 2024). SBSOD scores (Hegarty et al., 2002) also significantly correlated with ratings of pointing confidence, which showed that self-perceived spatial ability reliably transfers across settings. This offers practical implications for training simulations and could help identify individuals who would benefit from added support or adaptive guidance in high-stakes navigation. These results emphasize the need to distinguish between stable traits and dynamic states when designing training simulations, navigation systems, and safety protocols. They also suggest that navigation needs are dynamic and change depending on the scenario and the individual, and that simple wayfinding cues that guide individuals from point A to point B are not always useful. In safety–critical scenarios, it is important to have cues that are able to convey clear and visually intuitive information about dangerous areas quickly, so that individuals can make confident, informed decisions that keep them safe.

### Limitations and future work

The current study provided key insights about how virtual navigation cues may influence safety and spatial awareness. However, there are several limitations that should be acknowledged. One limitation is that the VE used in the current study was not cluttered and lacked dynamic elements typical of the real world. The absence of pedestrians, vehicles, and other urban distractions may have made navigation easier than it would have been if these items were present in an actual emergency. However, the current study did establish a reliable framework for testing cues that signal danger, and future studies could build on this work to evaluate cue effectiveness in more dynamic settings. This could introduce greater variability in navigation behavior and enable a more thorough evaluation of cue effectiveness under realistic, dynamic conditions.

Additionally, our current spatial memory task may not have effectively captured survey spatial knowledge, given the limitations discussed in the prior section, and instead tested participants’ working memory for where threat locations were. Future studies should consider adding additional metrics (e.g., a route recall task at the end of the study) to better test spatial memory.

The penalty for entering the danger zone also did not provide the same visceral consequence as a real-world situation would, and participants were made aware during the practice trials that there would be a danger zone along each route. This could have primed participants to avoid the danger zones regardless of cue type. We note also that in the current study, participants were limited to a set speed of 3 m/s and could not run, jump, or duck for cover. In a real-world setting, individuals would be able to adjust their speed or duck to avoid danger or to move through it more quickly. This flexibility of behavior may also impact cue use as well as preferred cues for safe navigation in the real world. Future work should test the MM and GA virtual cues in either ambulatory VR or in a real-world AR walking task outdoors, to assess how effort and cognitive load may affect the performance of the virtual cues.

Additionally, we cannot fully disentangle whether the observed affective states were due to the threat itself or due to the presence of the cues and their designs. The cues themselves may have induced anxiety, given that the cues were the only indicators of where the threat and danger zones were located. This means that the cues may have served as both navigational aids and the primary source of any anxiety experienced by participants during trials. If the cues are inducing additional anxiety during navigation, they could lead to a decreased ability to orient in the environment and to plan routes (Varshney et al., 2024), leading to inefficient navigation and increased risk of danger in a safety–critical scenario. However, it is important to note that the MM cue led to lower state anxiety ratings. These lower ratings may have been due to the MM cue conveying the full extent of the threat in the environment at one time, which allowed participants to preplan and see alternative routes, unlike the GA cue. Further work could investigate a scenario with a threat in the environment that is not conveyed directly through cues, such as the danger being signaled by an auditory gunshot, to aid in better understanding what is specifically producing anxiety for participants.

The current study also used the entire FOV of the Varjo VR-3 headset, which differs significantly from the FOV of current AR displays. Given that AR and mobile AR applications will likely be the media with which the real-world implementations of these navigation aids occur, future studies should evaluate whether the same cues remain effective under AR-specific constraints like limited FOV. These cues should be tested to evaluate whether current technology and hardware have enough computational power for these specific real-time overlays in mixed-reality environments.

Finally, our sample was not gender-balanced, and we utilized a convenience sample from the undergraduate University of Utah Psychology department, which resulted in more females being recruited to participate in the study than males. This may have limited the generalizability of our results. However, despite a difference in the number of males and females in our sample, we still observed some gender effects. Future work should recruit a more gender-balanced sample to further examine how gender differences affect cue engagement and affective responses. Overall, testing more balanced samples could improve the inclusivity and applicability of navigation systems in the future and ensure that the virtual cues being developed are effective for a wide range of users. The limitations discussed above highlight opportunities for future studies to extend this work by integrating more real-world complexity, emergent threats, and participant diversity.

## Conclusion

We evaluated the effectiveness of two virtual cues in supporting safe navigation and spatial knowledge development during a safety–critical scenario. Unlike traditional wayfinding tasks, participants could navigate freely within a virtual environment to reach the target beacon safely and efficiently. The results showed that both virtual cues were effective in reducing time spent in danger zones. However, the minimap cue lowered the state spatial anxiety that participants experienced during wayfinding. This study establishes a potential framework for integrating safety-focused virtual cues into future AR navigation systems that not only assess effective wayfinding, but also the impact of the cueing and situation on participants’ emotional reactions and spatial knowledge, which are known to affect navigation efficiency and success.

## Data Availability

The data collected and analyzed for this study are available from the corresponding author on reasonable request.
